# Procuring local net zero investment: A UK case study

**DOI:** 10.1007/s12053-025-10346-w

**Published:** 2025-07-01

**Authors:** Colin Nolden, Sean Fox, Emilia Melville, Katherine Sugar, Tedd Moya Mose, Caroline Bird, Jack Nicholls

**Affiliations:** 1 University of Sheffield, Sheffield, UK; 2 UK Energy Research Centre, London, UK; 3 University of Bristol, Bristol, UK; 4 University of Manchester, Manchester, UK

**Keywords:** Procurement, Net zero, Energy services, Contracting, Risk, Climate finance

## Abstract

Following over a decade and a half of austerity measures, and with costs of delivering statutory duties soaring, UK local authorities’ resources and capabilities to deliver net zero are diminishing. Decarbonisation funding provided by central government, meanwhile, is awarded competitively. To secure long-term, place-based net zero investments under these unfavourable circumstances, UK local authorities are increasingly turning to public procurement. A prominent example is Bristol City Leap, a Joint Venture Company procured by Bristol City Council between 2018 and 2022 to deliver around £1bn of investment in energy infrastructure and service delivery over 20 years through a concession agreement. Drawing on workshops and interviews with key stakeholders and experts, this paper examines the risks and opportunities of procurement and early-stage delivery of this public–private-partnership model. Using insights from transaction cost economics, it finds that this agreement has significantly increased net zero investment in return for increased risk and transaction costs. To ensure successful, just, and equitable delivery of promised place-based net zero investments, significant procurement capabilities, careful due diligence procedures, continuing institutional oversight, and independent measurement and verification are required.

## Introduction

Local authorities, and cities in particular, have a key role to play in supporting and delivering net zero given their governing responsibilities, their (albeit limited) tax raising and spending capacity, and their involvement shaping in energy demand practices (Barr et al., 2018; Betsill & Bulkeley, 2003; Bulkeley, 2010; LGA, 2021; NAO, 2021; Sugar et al., 2022). In the UK, however, local authorities are confronted with declining in-house capabilities due to cuts in central government funding, council tax freezes, and other rule changes which saw UK local authorities’ spending power fall by around a quarter in real terms between 2010/11 and 2019/20 (Green Alliance, 2020; Ogden et al., 2021). As a result, three local authorities found projected income no longer sufficient to meet projected spending in 2023 alone (Sandford & Brien, 2024). This implies that over half of the required local net zero investment of around £544bn will need to come from private sources (Innovate UK and GFI, 2022; Sugar, 2022; Innovate UK and PwC, 2023).

Of the 372 local authorities in the UK, meanwhile, over 300 have declared climate emergencies accompanied by net-zero objectives with target dates as early as 2025 (Nolden et al., 2024). Given the abovementioned shortage of funding, an estimated 95 per cent of local authorities require more financial resources to deliver their climate change strategies (LGA, 2021). Furthermore, government support for net zero delivery is mainly awarded through competitive processes that force local authorities to divert scarce resources to bidding processes with uncertain outcomes (NAO, 2021; Innovate UK and GFI, 2022; Nolden et al., 2024). As a result, innovative solutions are sought to address this funding, capacity, and capability shortfall (Innovate UK and PwC, 2023).

One area receiving increasing attention is public procurement which totals over £300bn a year in the UK and around €1.7trn across Europe (Sugar et al., 2022). In the UK, public procurement has a long history of being used to crowd in private investment by providing a stable stream of demand for a range of services, ranging from health care and education to energy and mobility (Lewis, 2021; Sugar et al., 2022). Outsourcing public services through procurement, however, gained notoriety in the 2000 s due to funding misallocation and commercial business models lowering the quality of public services in the pursuit of profit, with public–private-partnerships (PPPs) and Private Finance Initiatives (PFIs) as they were known as in the 1990 s receiving a particularly bad reputation in this context (Lewis, 2021).

Regarding decarbonisation, on the other hand, publicly procured outsourcing solutions have been less controversial, especially in the context of energy service contracting with its successful track record in improving energy efficiency, lowering costs, and contributing to emission reduction targets (Sorrell, 2007; Nolden et al., 2016; Polzin et al., 2016; Keegan, 2018; Tingey & Webb, 2020; Gillham et al., 2023). While in-house solutions are generally preferable to retain public capabilities and avoid profiteering, outsourced ‘modes of governance’, such as relational and long-term contracts, have been successfully procured to deliver energy service improvements (Polzin et al., 2016; Sorrell, 2007; Tingey & Webb, 2020). Such contracts involve different degrees of local authority control and risk, and different transaction and production costs, and hence ability to crowd in private finance (Bradach & Eccles, 1989; Sorrell, 2007; Nolden et al., 2016; Polzin et al., 2016; Keegan, 2018).

Procurement frameworks such as Refit and the Carbon and Energy Fund (CEF) are the main drivers of such solutions (Nolden and Sorrell, 2016; Keegan, 2018; Tingey & Webb, 2020; Sugar et al., 2022; Gilham et al., 2023). By lowering transaction costs, these procurement frameworks facilitate energy service contracting for individual public sector sites (such as a hospital), multiple buildings with similar uses (such as schools), or specific technologies (such as heat networks) (Nolden et al., 2016; Polzin et al., 2016). The total value of the UK energy service contracting market has been estimated at $ 115 m in 2017/18 although market size is notoriously difficult to analyse (Nolden and Sorrell, 2016; Keegan, 2018). Despite the long history of energy service contracting in the UK, however, such arrangements have not been used to increase energy infrastructure investment and service delivery at a city-scale. The biggest risk lies in energy service companies (ESCOs) cherry-picking profitable projects and leaving higher-risk, longer-term, and less-monetisable projects to the local authority.

With shrinking budgets and declining in-house capabilities for net zero delivery, such solutions are receiving growing interest among UK local authorities (HM Government, 2023; Sugar et al., 2022). To shed light on associated risks and opportunities, we examine Bristol City Leap (BCL), a 20-year concession agreement as a case study contract combining quick-win/high-return and low-return/long-term projects (BCC, 2022a, 2022b; HM Government, 2023). This agreement commits Ameresco Ltd (an ESCO operating across the US, Canada, and Europe) together with Vattenfall UK (a Swedish government owned energy supplier and district heating system operator) as an essential subcontractor to invest around £424m in the first five years (which has since increased to £527m with investments forecast for the first six years up to 2029 revised to £771m) with a total investment volume of nearly £1bn over 20 years to deliver a series of Key Performance Indicators (KPIs; see Annex 1; BCL, 2022, 2024; Nolden et al., 2023). The research question is as follows:

What are the risks and opportunities of procuring city-wide energy infrastructure investment and service delivery contracts such as Bristol City Leap?

We answer this question by using insights from transaction cost economics (TCE), a framework commonly used to assess the economics of energy service contracting (Nolden et al., 2016, 2025; Polzin et al., 2016; Sorrell, 2007). This paper is structured as follows: Section 2 introduces the analytical framework and the methodology. Section 3 analyses the BCL model using insights from transaction cost economics. Section 4 discusses the trade-offs which need to be taken into account in procuring such a solution. Section 5 concludes.

## Methodology

### Background

To establish the nature and success of public–private-partnerships in delivering energy services we undertook a Scopus review. A Scopus search (ALL ("public private"AND"transaction costs"AND"energy servic*")) identified 28 papers. After screening their abstracts, 19 were identified as relevant, of which three are books, which left 16 papers for review (Table 1).

**Table 1:** Comparison of existing studies the transaction costs involved in energy service delivery through public–private partnerships

	Transaction costs	TCE	PPP	Single technology	Single site	Aggregation
Ahmadi et al. (2020)	X			X		
Bougrain (2012)	X		X	X		
Carbonara and Pellegrino (2018)	X		X	X		
Fecondo and Moca (2015)	X		X	X		
Ghiani et al. (2022)						X
Krøtel (2015)	X					X
Martinello et al. (2020)			X	X		
Marques and Geddes (2019)			X	X		
Nour El-Din (2024)	X			X		
Pardo-Bosch (2019)	X		X		X	
Polzin et al. (2016)		X	X	X		
Selviaridis and Wynstra (2015)		X		X	X	X
Shakeel et al. (2024)	X			X		
Spyrdiaki et al. (2016)	X				X	
Tahir et al. (2024)			X	X		
Wan et al. (2023)	X				X	

This review found no paper using a UK-related case study. Furthermore, most papers focus on single technologies, such as street lighting which is analysed in Germany (Polzin et al., 2016), Italy (Fecondo & Moca, 2015), the USA (Marques & Geddes, 2019) and energy from waste which is analysed in a European context (Martinello et al., 2020) and the UK and Canada (Tahir et al., 2024). Only three papers focus on aggregation as opposed to technology-specific or site-specific solutions (Ghiani et al., 2022; Krøtel, 2015; Selviaridis & Wynstra, 2015) and none looking at city-wide aggregation. While all these papers engage with transaction costs to some degree, only two papers employ transaction cost economics in their analysis (Polzin et al., 2016; Selviaridis & Wynstra, 2015).

Overall, these papers tend to take a favourable view of PPPs in general and their applicability to energy services specifically, especially in the context of single technologies. For example, Fecondo and Moca (2015: 231) suggest that PPPs allow “governments to achieve their [energy efficiency] targets with only a fraction of the public funding that would otherwise be required, with the private sector taking on both the financial and performance risks”. Marques and Geddes (2019: 324), meanwhile, suggest that “the PPP option has a great potential with several strong points and with manageable weaknesses”. What is lacking is an evaluation of the benefits and shortfalls of PPP arrangements for the delivery of complex energy infrastructure investment and service delivery projects involving multiple technologies and sites on a city-scale.

### Analytical framework

This paper uses insights from transaction costs to address this shortfall by embedding the ‘mode of governance’ underpinning BCL in the wider framework of procuring innovative energy infrastructure and service delivery solutions on a city-scale. The ‘mode of governance’ ranges between in-house provision and various degrees of outsourced provision of good and services (Williamson, 1993; Furubotn & Richter, 1997; Rindfleisch & Heide, 1997; Sorrell, 2007; Nolden et al., 2016; Gillham et al., 2023). In the context of net zero energy service infrastructure delivery at local authority level, the ‘mode of governance’ depends on their in-house capacity to organise the delivery, the scale and depth of infrastructure investment sought, and the availability of technical assistance and intermediation (Williamson, 1993; Sorrell, 2007; Nolden et al., 2016; Polzin et al., 2016; Tingey & Webb, 2020; Sugar et al., 2022; Gilham et al. 2023). ‘Modes of governance’ are often portrayed as a spectrum between fully integrated in-house hierarchies and fully outsourced and unbundled markets (Table 2; Williamson, 1993; Pint & Baldwin, 1997; Polzin et al., 2016; Marques & Geddes, 2019).

**Table 2:** Spectrum of governance structures for local authority net zero delivery (adapted form Marques & Geddes, 2019; Pint & Baldwin, 1997; Polzin et al., 2016)

**Governance**	**Hierarchies**				**Markets**
Contracts	Vertical Integration	Relational contracts	Long-term contacts	Short-term contracts	Spot market
Energy service contracts	In-house management of energy services	Municipal Utility Company	Energy Service Company	Energy service arrangement for an event	Flexibility service provision

In the context of energy services, however, short-term contracts are confined to events while spot markets (to the right of Table 2) are confined to the provision of flexibility services. Choosing a ‘mode of governance’ is determined by the ability of a contractual agreement to lower (anticipated) total costs, which are the sum of *production cost savings* and *transaction costs*. In an outsourcing agreement one would expect both to increase, with viability hinging on increases in *transaction costs* not outweighing increases in *production cost savings* (Williamson, 1993; Sorrell, 2007; Polzin et al., 2016).

Organisations delivering energy services can lower such *production costs* through *economies of specialisation, scale, and learning* (Table 3; Williamson, 1993; Sorrell, 2007):

**Table 3:** Transaction economies associated with the procurement of energy infrastructure investments and service delivery

Transaction economies	Example	Source
Economies of specialisation	Institutional capabilities to deploy and operate a district heating system	(Williamson, 1993; Sorrell, 2007)
Economies of scale	Sharing knowledge, skills and purchasing power across projects	(Williamson, 1993; Sorrell, 2007)
Economies of learning	Cumulative experience of deploying and operating multiple projects	(Williamson, 1993; Sorrell, 2007)

In this context of a 20-year concession agreement to transform infrastructure alongside the delivery of energy services, viability hinges upon increases in *transaction costs* not outweighing total *investment* and, as we will later see, the *social value* created throughout the contractual period, rather than just *production cost savings* (BCC, 2022a, 2022b; BCL, 2024; Sorrell, 2007). Such *transaction costs* comprise monetary, time, and inconvenience expenses incurred by both the procuring authority and potential service providers resulting from preparing, negotiating, establishing, executing, monitoring, and enforcing the associated contract (Table 4; Furubotn & Richter, 1997; Rindfleisch & Heide, 1997; Sorrell, 2007; Vining & Globerman, 1999). These include:

**Table 4:** Transaction costs associated with the procurement of energy infrastructure investments and service delivery

Transaction costs	Example	Source
Search costs	Tendering, identifying a potential client or contractor, verifying their suitability, preparing and evaluating bids and selecting a preferred contracting partner	(Nolden et al., 2016; Sorrell, 2007)
Bargaining costs	Negotiating and preparing the contract, monitoring contract performance, enforcing compliance, negotiating changes to the contract when unforeseen circumstances arise and resolving disputes	(Nolden et al., 2016; Sorrell, 2007)
Opportunism costs	Associated with either party acting in bad faith	(Nolden et al., 2016; Sorrell, 2007)

*Transaction costs*, in turn, are influenced by transaction attributes and underlying risk (Bradach & Eccles, 1989; Sorrell, 2007). To determine the suitability of a particular ‘mode of governance’, it is therefore necessary to first consider how transaction attributes (Table 5) influence *transaction costs* (Nolden et al., 2016; Polzin et al., 2016; Sorrell, 2007). The following transaction attributes are of relevance in this context: Technical asset specificity, human capital specificity, uncertainty, dedicated resources, task complexity, market competitiveness, and  the institutional framework (see Table 5).

**Table 5:** Transaction attributes associated with the procurement of energy infrastructure investments and service delivery

Transaction attributes	Example	Source
Technical asset specificity	Specialised and embedded equipment such as a district heating system	(Pint & Baldwin, 1997; Toffell, 2002; Polzin et al., 2016)
Human capability specificity	Dedicated skills for heat pump installation and adjustment	(Pint & Baldwin, 1997; Toffell, 2002; Polzin et al., 2016)
Uncertainty	Regarding costs and profit margins of infrastructure investments	(Polzin et al., 2016)
Dedicated resources	Specialisation in developing and operating district heating networks	(Pint & Baldwin, 1997; Toffell, 2002; Polzin et al., 2016)
Task complexity	Information asymmetry regarding risks of operating a district heating network	(Sorrell, 2007; Polzin et al., 2016)
Market competitiveness	Pricing close to the marginal cost	(Sorrell, 2007; Polzin et al., 2016)
Institutional framework	Regulatory requirements and resource provision	(Sorrell, 2007; Polzin et al., 2016)

Finally, this paper also takes into account the risk associated with this ‘mode of governance’ given the need to ensure just outcomes in the context of public procurement and public service delivery (Chiles & McMackin, 1996). Specifically, it considers the risk of contractors prioritising areas of investment with high returns (such as electricity generation on public sector land) to the detriment of those with lower returns (such as demand reduction across social housing). The latter could for example include interventions in disadvantaged neighbourhoods associated with high transaction costs as a result of high *human capability specificity*, a need for significant *dedicated resources*, and high *task complexity*, yet deliver significant *social value* (Garvey et al., 2022, 2023; Gillham et al., 2023).

### Data collection and analysis

The procurement and delivery of BCL was analysed using a qualitative methodology. This encompassed two workshops, regular meetings with BCL representatives, and interviews with a total of 14 individuals between July 2022 and February 2025 (Table 6). During the procurement phase we only interviewed representatives from BCC and BCL, with several BCL representatives having moved there from BCC, Ameresco, and Vattenfall. During early-stage delivery, interviews were conducted with representatives from external organisations who provide independent perspectives on the benefits and shortfalls of BCL. Regarding specific procurement issues, few interviewees provided specific evidence given the commercially sensitive nature of such information. Four interviewees also opted out of recording due to commercial confidentiality concerns. Commercial confidentiality also restricted access to relevant documents, despite a Non-Disclosure Agreement in place between BCC, BCL and the University of Bristol, where this research was ethically approved, and in which name it was undertaken.

**Table 6:** List of research participants

Number	Recorded	Organisation	Date
#1	n/a	Bristol City Council	14/07/2022
#2	n/a	Bristol City Council	14/07/2022
#3	Recorded	Bristol City Leap	25/05/2023
#4	Recorded	Bristol City Council	25/05/2023
#5	Recorded	Bristol City Leap	22/06/2023
#6	Recorded	Bristol City Leap	10/07/2023
#7	n/a	Bristol City Leap	15/11/2023
#8	n/a	Bristol City Leap	15/11/2023
#9	Recorded	Bristol City Leap	31/01/2024
#10	Recorded	Bristol City Council	26/03/2024
#11	Recorded	Former ESCO and public sector	26/01/2025
#12	Recorded	Financial advisor	05/02/2025
#13	Recorded	Measurement and Verification expert	07/02/2025
#14	Recorded	Business model advisor	11/02/2025

While this is disappointing, and lowers the depth of insight, the evidence provided here is new and closes a knowledge gap on how the transaction costs, risks, and rewards of place-based energy infrastructure investment and service agreements are managed in their procurement and early-stage delivery. In future, freedom of information requests might reveal deeper insight and complement this research. The interviews, meetings, and workshops followed a process approved by the Law Ethics Research Committee of the University of Bristol. Following the provision of an information sheet and the completion of a written consent form, interviewees were asked tailored semi-structured questions according to their embeddedness in the contracting process.

Ten of the interviews were recorded and subsequently transcribed for analysis in NVivo. Coding was undertaken iteratively, following a double coding process to minimise researcher bias and improve the robustness of our analysis. Recorded interviews #3-#6 were coded using parent nodes ‘Contract’, ‘Contractor’, ‘Constraints’, ‘Drivers’, ‘Externals’ and ‘Investment’. Recorded interviews #9 and #10 were coded using parent codes ‘Externals’, ‘Finance’, ‘Policy – asks’, ‘Policy – national’, ‘Policy – local’, ‘Priority area’ as they stem from a different research project. Finally, recorded interviews #11-#14 were coded using parent codes ‘Government’, ‘Modes of governance’, ‘Procurement criteria’, ‘Procurement frameworks’, ‘Scrutiny’, and ‘Transaction costs’.

For this paper, quotes from relevant parent nodes were allocated to new nodes relating to this investigation: ‘transaction attributes’, ‘transaction costs’, ‘risks’, and ‘delivery and scrutiny’. Data will be made available on request upon publication. The following section summarises the results of interview analysis.

## Findings

### Assessing production cost savings

The City of Bristol has a longstanding reputation as a centre for green innovation and environmental activism. It was awarded the status of European Green Capital in 2015; was the first UK council to declare a climate emergency in 2018; and pioneered the integration of the UN Sustainable Development Goals into local plans and monitoring activities (Fox and Macleod, 2023). However, like all UK authorities it has experienced a period of protracted austerity beginning in 2010 (Sugar et al., 2022). The impact of austerity was significantly amplified by an increasingly competitive infrastructure funding context at national level (Innovate UK and GFI, 2022):

*“I think the nature of government funding as you well know now is rapidly becoming more of a competitive environment, it’s not a guarantee that you’ve delivered a good project before that you’ll get funding next time around, it’s more of a kind of Hunger Games”* (#5)

*“Councils compete against each other, it’s absolutely crazy. […] it’s about who gets there first, not the best bid”* (#7)

*“The public sector has been stripped of capability and stripped of the ability to spend money and needs access to both those things”* (#11)

An unintended consequence of this competitive approach is that demand for activity surges upon funding allocation with not enough time to scrutinise contractors appropriately nor to progress strategically. A good example is the Public Sector Decarbonisation Scheme (PSDS) where “*government set the time scale so short that the public sector was forced into the arms of contractors [without] the time to work out what [the] project portfolio could be*” (#11). This has resulted in BCC being quoted at £400k for a decarbonisation project before the competitive allocation of PSDS funding, and £750k after the award due to the associated peak in demand across the public sector (#7 and #8).

BCC has nevertheless done comparatively well in this competitive environment, having secured the largest share of Heat Network Delivery Unit (HNDU) funding (£1.3m out of a total of £33.8m; DESNZ, 2023). In total, BCC has invested around £100m into decarbonisation over a ten-year period (BCC, 2022a, 2022b; BCL, 2022). While this sum is impressive, it is nowhere near the £9bn capital investment required to decarbonise the city’s heat and transport infrastructure (BCC, 2021). To attract investment into the city to help it achieve its decarbonisation targets, and since 2021 its 2030 net zero target, Bristol City Council (BCC) published the City Leap Prospectus in 2018 (BCC, 2018).

It was based on the premise that BCC controls around 17% of the 27,500 houses in Bristol, 40% of the land, and its estate amounts to around 1% of the built environment. BCC thus recognised the requirement for a step change using its existing assets as leverage which led to the inception of City Leap (#1, #2). The Prospectus launched a soft market testing phase which cost around £500k and exceeded all expectations with approximately 180 expressions of interest returned “*from major multinational energy companies, financial institutions, institutional investors, technology companies right down to local community groups and individuals, a number of local supply chain companies* “ (#6). These revealed a desire for a ‘first right of refusal’ among suppliers while BCC’s risk profile revealed a preference for a joint venture company (JVCo) with one or more large suppliers to:

- Ensure the contractor takes on both risk and reward
- Create an outward facing entity which is unusual within conventional procurement arrangements
- Transfer BCC’s Energy Service team to the JVCo (when energy service is capitalised henceforth it refers to BCC’s Energy Service team)
- Sell the district heating network
- Award strategic control within the JVCo to minimise future political interference and allow it to operate beyond the constraints of public procurement legislation

Once this type of concession agreement alongside the scale and depth of energy infrastructure investments and service delivery had been clarified, BCC embarked on a procurement exercise through the Official Journal of the European Union (OJEU). Complexity associated with the structure of the agreement necessitated a combined dialogue and negotiation process as part of the Invitation to Participate (ITP) involving three parallel bids. At the same time, BCC needed to ensure transparency and fairness throughout, especially with regards to data management around more than 1,500 clarification questions (#1, #2). The winner Ameresco with Vattenfall as an essential subcontractor was announced in late 2022 and the contracts were signed in early 2023 (BCC, 2022a, 2022b, 2023). Their assumed ability to increase investments in energy infrastructure and associated services, while creating significant social value stems from the following economies:

*Specialisation economies*: The delivery partners’ business focus on energy infrastructure investment and service delivery lowers their production costs vis-à-vis the local authority whose primary focus lies elsewhere, even if they employ a large energy service team.

*“I think Bristol’s done very well … an exemplar really in terms of budget allocation but … it’s not ever going to be enough. You know the scale of the challenge is such that even Bristol City Leap’s not going to be enough to decarbonise the whole city”* (#5)

“*The heat network is going to require hundreds of millions of pounds over the next 10, 15 years to build out, and then you’re relying on making sufficient connections… you know over a 40 plus year time horizon to make a return on that investment. That’s not a good place for a local authority to be*.” (#6)

*Scale economies*: The delivery partners’ experience in delivering projects across multiple scales and sites with multiple clients implies that they benefit from greater technical, commercial, legal, and managerial expertise in energy service delivery than their clients. They might also benefit from preferential access to finance and equipment.

*“It might be that an individual project is several hundred million, plus an operations and maintenance phase of 15, 20 years, and we’re comfortable with those kind of deals. [BCL] is slightly different because the billion pounds … is the project capital, but there’s still the taking care of it, and there’s operations and maintenance and things for the assets that go in, which potentially need doing.”* (#3)

*“[BCC] does not offer the scale that’s needed to attract the money that we know is out there. Do we have the capacity to get things ready … actually get that market ready? Right, we don’t have the expertise.”* (#4)

*“The difficulty with the sort of roll out of distributed networks, be it heat or power, it needs somebody to put in the investment in the infrastructure. It never works for a single client to do that.”* (#11)

*Learning economies*: The delivery partners’ experience combined with the duration of the contract enables them to share knowledge across contracts, carry forward lessons from one to another, and operate less dependent on public sector budget cycles.

*“[BCL] is a really exciting opportunity to step in and do it even more over a much longer period of time. [Ameresco] often signs long term contracts, so a lot of [their] energy performance contracts […] have a long tail.* (#3)

*“[The concession agreement is] 20 years, that's much more sort of long term and you know, you don't really get the funding pots that match that.”* (#10)

By benefiting from all these economies, and by dedicating assets and learning capabilities to maintaining and enhancing these economic advantages, Ameresco and Vattenfall, through BCL, appear to be well placed to increase energy infrastructure *investment* and energy service delivery across Bristol in pursuit of BCC’s net zero target. Anticipated increases in *investment* relative to the risk of opting for this ‘mode of governance’ are elaborated in the following section.

### Investments vs risk

BCC’s net zero *investments* amounted to £18.5m/a on average between 2017 and 2022 (BCC, 2021, 2022a). A similar level of *investment* is planned by BCC in the coming years (BCC, 2022a, 2022b, 2023). BCL, according to its original business plan and KPIs (see Appendix 1), anticipates *investment* of around £125m/a on average until 2028 (BCC, 2022a). This would present a 6.5-fold increase in net zero *investment* in the period 2023 to 2028 compared to the reference period 2017 to 2022 and BCC’s planned *investments*. According to the revised business plan, BCL anticipates average *investment* of around £150m/a until 2029, which would represent an eightfold increase (BCL, 2024).

While these figures sound impressive, it is unclear exactly where this *investment* will be sourced from and how BCL will “*get away from that privatisation of profit and nationalisation if you like of risk which generally public private partnerships are seen to be… within our world*” (#11). The c£86.7m earmarked for renewable energy generation according to the KPIs (Annex 1) is attractive for private finance thanks to solid returns on *investment* while the c£83.7m earmarked for energy efficiency *investment* to reduce demand and optimise electrification of heat solutions are associate with lower, if any, returns (Nolden et al., 2016; Innovate UK and PwC, 2023). It is much more difficult to attract private finance into such demand-side solutions, so the overall burden lies with public finance if demand-side solutions are not explicitly cross-subsidised through more profitable supply-side solutions within the contract.

*“There's risk for the private sector that if you are delivering projects at a lower margin, hoping that you'll win the projects that are higher margin later and vice versa, if you're the public sector hoping that your contractor will deliver some of the better saving projects later. […] So, you do need to set that out at the beginning I think as to what success looks like for both sides and can you find a nice breakeven point where you both get the thing that you need. It isn't about winning in that way. It can't be about ripping each other off for these bigger projects on these longer concessions”* (#11)

BCC and BCL interviewees, meanwhile, are confident that this concession agreements with its KPIs, rather than specific objectives, provides enough flexibility to incentivise innovative *investment* in energy infrastructure and service delivery:

“*I mean you couldn’t list out all the measures in every building and say this is what you’re bidding, how much is that … we’ve got an idea … but it will shift over time with technology changes, buildings come in and out, assets change, you know energy prices change – all sorts of things. But that’s fine, because in the mix of it we can crack on and craft those projects, or batches of projects to fit within the concession and get on with them*” (#3).

“*If the council was to say: ‘that tower block over there, we want to completely reclad it, we want to take every flat within that tower block up to an EPC rating of B or higher’. Rather than going through a procurement process which would [take years] and even then I guess not having a sure outcome in terms of the quality of the work that’ll come out of it, you know costs and how much that could impact on..value for money.. for a council. Rather than doing that, the council has a pre-procured partner in City Leap… ready to deliver*” (#5).

*“We needed to try and avoid being overly prescriptive and allow City Leap to innovate in terms of what they could respond back with. And I guess those were the key messages. […] [BCC] quickly arrived at the conclusion that you know the strategic part of City Leap was likely to be quite a major organisation rather than a really small one for example” (#6)*

*“City Leap… is not a straightforward contract, you know… We're not getting a roof repaired on the building, where it's like the beginning and end and that's that… It's very much a partnership, it's a very long-term agreement…. Its aims are around carbon neutrality”* (#9)

While this concession agreement appears to lower the risk of developing innovative solutions, more risk is borne by the local authority compared to conventional energy service contracts targeting individual technologies or sites due to *opportunism* risks and *legitimacy* concerns associated with unprecedented energy infrastructure *investments*.

*“Two key concerns were around value for money and making sure […] the council wasn’t over a barrel having to pay well over the odds for projects, or where it was making contributions. And also, that City Leap remained true to what it was trying to do, which was a strategic approach to decarbonisation, and didn’t end up with the private sector just […] cherry picking it essentially. So you know that’s manifest itself through the business plan, and as City Leap now we have to demonstrate that there are a range of projects being delivered across a number of technologies, not just say we’re only going to do heat networks for the next 20 years.”* (#6)

To ensure the embedding of BCC’s principles into BCL, a near wholesale transition of BCC’s Energy Service team into BCL took place with 27 of the 35 former BCC staff transferring through a TUPE (Transfer of Undertakings (Protection of Employment)) transfer when BCL had been procured, and a few choosing not to. BCC still employs 5 people in its remaining Energy Service team with BCC’s client function overseeing the delivery of the BCL contract (#7 and #8). This has been a particularly controversial aspect of the contract alongside the sale of the heat network at cost price in the concession agreement. Few councils in the UK have experience developing and managing large-scale heat network expansion and BCC made the decision to retreat from running and expanding the heat network because it carried significant risks and was not a core statutory duty (see quote above on the + £100m investment required for its development).

These transfers ultimately constituted key incentives alongside the ‘right of first refusal’ to attract commercial interest. Granting the private partner the ‘right of first refusal’ on BCC energy infrastructure contracts over the next 20 years accelerates the development process by reducing risks and lowering *transaction costs* associated with repeat procurement. Importantly, this contractual agreement does not force BCL into accepting every infrastructure and service delivery contract opportunity commissioned by BCC. Instead, it allows them to model a predictable revenue stream including projects that may not have been profitable had they been tendered through a competitive procurement process.

*“[BCL has] the ‘first right of refusal’ to develop the projects if they relate to energy and carbon saving within the estate of Bristol – which is the thing, like that’s the thing that gets us in and gets us established, and allows us to build our capability in the city, and then go to other organisations as well and spread that capability and expertise to help everyone decarbonise*” (#3)

*“[BCL has] the ‘first right of refusal’ to carry out all low carbon energy infrastructure projects on the council’s estate. So that is the heart of City Leap… if someone else was replicating this elsewhere they would have to do something similar. From the private sector’s perspective, it’s like ‘okay I can understand there’s an opportunity there, I can see how I’m going to make a return on the investment I’m going to make in going through the procurement process’, because it was expensive, fairly expensive”* (#6)

As an incentive, the ‘right of first refusal’ builds on BCC’s track record of financially credible projects. This track record demonstrated an attractive policy and *investment* environment, favourably viewed by commercial finance organisations (as was clear from the number of initial bids received). Yet this quasi-monopolisation inherent in the ‘right of first refusal’ increased the challenge risk during the procurement process, which cost BCC £9m to procure (with some sources suggesting £11m), while increasing the risk of underdelivery of challenging demand-side projects.

*“First refusal on the delivery… leads to all kinds of shenanigans going on and it does unfortunately lead to the cherry picking of projects that might deliver the best return for the private sector rather than the best savings for the public sector”* (#11)

Unsurprisingly, the threat of a judicial review accompanied the procurement process from start to finish, which started outcome-based, then broadened out, before it was narrowed down into a set of evaluation criteria. Work was front loaded due to high bid costs and the nature of this one-off opportunity (#1 and #2). To complete the assessment of BCL, anticipated *investments* and the *risk* associated with this ‘mode of governance’ need to be compared to *transaction costs* (Nolden et al., 2016; Sorrell, 2007).

### Transaction costs

Keeping *transaction costs* low in procuring BCL is linked to BCC’s leadership in climate action and the significant capabilities of its Energy Service team (35 people before the TUPE transfer) compared to the size of the city. The *transaction costs* of procuring BCL, and their mitigation in the contract negotiation process, are as follows:

*Search costs* were lowered in the assessment of suitable delivery partners by procuring external expertise:

*“We got some sort of commercial expertise in at that point just to kind of kick the ideas around with us to make sure that actually the kind of proposals that we were taking forward to cabinet were grounded in reality and a good kind of business sense really.”* (#5)

*“We had both commercial and legal advisors sat alongside us, so we were able to get their input in terms of coming up with options, which we could then take back and discuss with the board that we had in place... And we needed that to be able to have the sort of discussions that we needed to have, because they’re strategic and risk based”* (#6)

Bringing in commercial and legal expertise helped establish the necessary concentration of expertise to compile, filter, and verify relevant information to lower the *search costs* associated with the procurement exercise. Simultaneously comparing these multiple bids also lowered the costs of appraising offers while incentivising pricing close the marginal cost of energy infrastructure investment and service delivery through *market competition*.

*Bargaining costs* were lowered through the establishment of performance benchmarks in the form of KPIs (Annex 1) based on the pipeline of historic projects developed by BCC’s Energy Services team, BCC’s experience of setting up and winding down an energy supply company (Bristol Energy), and their appetite for future initiatives, driven by public policy commitments:

*“Part of the value of Bristol Energy at the time was actually our ability to bring eyes to City Leap”* (#4)

*“Bristol was in a fortunate position of having that Energy Service and people on its side of the table who spoke the same language essentially, because that’s the way the Energy Service was run, because it had been doing projects over a number of years that made a return on investment and had also kicked off the heat network”* (#5)

The Energy Service’s experience in delivering projects lowered *uncertainty* regarding the cost and revenue structure of energy infrastructure investment and service delivery projects as well as related contracts and associated *task complexity* as technological potentials are known, which lowers information asymmetries.

*Opportunism costs* were lowered through BCC’s long-term experience in data accumulation, benchmarking, and the establishment of baselines in relation to energy, social value, decarbonisation, and sustainability more broadly, and their monitoring over time (Fox and Macleod, 2023).

*“There’s a load of work that’s been done in Bristol separately by the council and others looking at the carbon footprint of the whole city, and their own estate and that kind of stuff”* (#3)

“*Time and effort went into putting the systems in place a long time ago to make sure the council had good data in general*” (#6)

The availability of data facilitated comparison across benchmarks, the definition of contract scope and depth, contract negotiations, access to finance, and measurement and verification (M&V), thereby lowering information asymmetries and associated *opportunism* risk.

While BCC is confident in its contract monitoring capabilities, balancing requirements of a ‘fair and transparent’ procurement process complicated the ‘negotiation’ stage of dialogue to an extent which would not exist in an unregulated environment (#1, #2). Despite these risks, several BCC and BCL representatives are confident that the concession agreement contains sufficient checks and balances to mitigate against *opportunism* while ensuring *legitimacy* over the 20-year duration.

*“So we’ll be around the table together, the council and City Leap. You know obviously we’ve got teams in there as well. So there’ll be a collective accountability within that to monitor that deal to make sure it’s performing. We’ve learnt a lot with our own companies as well around the performance […] it’s not like we’ve cast it off and then we watch it happen at a distance, it is an active partnership”* (#4)

*“A lot of time and thought went into how to hold the strategic partner to account – and there are real teeth in that contract.” #6).*

*“And the important thing is you've got teeth built into the contract in the first place”* (#9)

These findings suggest that BCC has gone to considerable lengths to minimise *transaction costs* and mitigate risk in the process of procuring BCL. However, while the procurement of this concession agreement was thus lengthy and expensive, the achievement of the KPIs, despite these reassurances, is by no means guaranteed.

### Delivery and scrutiny

Crucially, it is unclear whether BCL will indeed deliver across both quick-win/high-return and low-return/long-term projects while providing enough flexibility within the contract to adapt to changing circumstances, and how delivery will be scrutinised throughout.

*“There will be failings within BCL. Where we, meaning everybody falls down is that we rely too heavily on anecdote, and we rely too heavily on Ameresco and Bristol City Council are going to tell you that this is the greatest thing that's ever happened because of course they are. So where's the independent scrutiny coming in that says where did this actually fail?”* (#11)

Citing water and rail companies, another interviewee points towards the “*tension between the short-term profit maximisation of private sector companies versus the long-term profit maximisation that you want in long-term infrastructure assets*” (#14). On the other hand, a measurement and verification expert working on energy service contracts in Scotland suggests that “*60% to 70% of the projects we worked on did hit target and the rest didn’t but then either a shortfall has been made up [or] they didn’t get paid their final sort of milestone payment if you will*” (#13). This suggests that flexible systems of checks and balances (‘teeth’) within such concession agreements can acknowledge “*an error bar on each project but as long as we’re doing all of them we will get some savings here and we will lose out there*” (#11) while overarching KPIs are being delivered.

The key mechanism to ensure such an outcome within this concession agreement is its significant social value component alluded to above (Lazzarini, 2018). At 14.5% of project value, social value provides a means of holding Ameresco and Vattenfall to account while incentivising the delivery of both quick-win/high-return and low-return/long-term projects.

“*The key strength of the [social value] framework we've got is that actually we are able to [hold suppliers to account]…. We use approximate financial values as part of our mechanisms… This is what we get associated with compensation payments for non-delivery*” (#10)

*“Effectively, to put together the program in a smart way that effectively it's like if the smaller projects or lower return projects were delivering more social value you then set a social value requirement that meant you had to do the nice fat juicy project but also the social value projects in order to hit your metric and you can see that the private sector understands that, they would see that that's a legitimate ask. If the smaller projects, the ones with the social value were rubbish, were crap projects being done for vanity, that would be different. But they sort of get that the government or the public sector has multiple objectives they're trying to hit. In that sort of framework, I think you can get there.”* (#12)

Regular scrutiny of such outcomes is thus necessary to ensure that concession agreements which successfully attract private investment into city-wide net zero delivery achieve their KPIs and balance the abovementioned tension between short-term profit maximisation objectives of private investors and long-term investment requirements for a just transition to net zero.

## Discussion

Procuring city-wide investment is essential if cities want to contribute to net zero delivery. This is particularly relevant in the context diminishing financial resourcing and capabilities among local authorities which reduces their ability to deliver net zero in-house across their public sector estates, let alone the cities they govern and provide for. In the UK, where “*the Thatcherite vision of taking any power away from local authorities and putting it all into the private sector*” (#11) and austerity measures since the 2008/9 financial crisis have reduced local authority financial resourcing and capabilities by at least 25%, resourcing and capabilities for in-house delivery are particularly constrained (Green Alliance, 2020; Ogden et al., 2021; Sandford & Brien, 2024).

To attract investment into technology or site-specific energy service solutions, a range of contracting governance arrangements are being pursued by UK local authorities (Tingey & Webb, 2020). BCL marks a step change in scaling up such arrangements to the city-scale to help Bristol achieve its net zero target.

*“Anyone who's given themselves a 2030 or 2035 or 2040 [net zero] target needs to be getting on and doing stuff now. So, people are making the best of where they are. And also, there's pretty limited imagination sitting, there's nobody really pushing them to think super creatively. Bristol's been the best that we've seen in the sense of, what they did was have a mayor, and an ability to convene, get a really strong kind of conceptual community support for change, and a vehicle for making that change happen”* (#12)

While initial soft-market testing based on the prospectus was relatively cheap (*search costs*), both monetary and *transaction costs* were high during the procurement phase (*bargaining costs*) given the unprecedented nature and scale of energy infrastructure *investment* and service delivery sought. BCC needed to balance the need for *investment* and the expected returns this entails with the need to ensure value for money while maintaining *legitimacy* (“*not over a barrel*”). The total cost of procuring BCL amounted to around £9m (possibly £11m), a significant proportion of which on due diligence, often involving external expertise (“*commercial and legal advisors*”). Few local authorities in the UK are in a position to afford such expenditure on a procurement exercise for non-statutory services and this is a “*massive risk for [BCC] because opposition…were just circling…waiting for failure*” (#4). Yet at this stage of project delivery and business planning, the *investment* benefits of procuring BCL appear to outweigh the *transaction costs* over the 20-year concession agreement (Table 7):

**Table 7:** Transaction attributes, costs, economies, and risk of procurement and delivery of BCL

Transaction attributes	Transaction costs	Transaction economies	Transaction risks
Technical asset specificity/Human capability specificity	Insufficient BCC in-house capability to attract investment and deliver city-wide decarbonisation yet sufficient to procure complex procurement exercise	Ameresco and Vattenfall's significant technical asset and human capability specificity creates economies of scale, specialisation, and learning	Technical risk: Using waste heat from an incinerator might be classified as zero carbon heat in the current regulatory regime but future taxonomies might classify it as high carbon heat, thus requiring a contingency plan to shift to a lower carbon source of heat
Uncertainty	BCC had very good knowledge of costs and profit margins of infrastructure investments thanks to its strong track record, but it does not have the capabilities to increase energy infrastructure investment ten-fold	Ameresco and Vattenfall bring in their national and international expertise and supply chains to lower costs and increase their profit margins of energy infrastructure investments	Legitimacy risk: if BCL is perceived as cherry-picking profitable projects to the detriment of more complex projects, and if social value generation falls short of agreed on KPIs, net zero delivery at a local level using such a ‘mode of governance’ might fall out of favour
Dedicated resources	While the BCC Energy Service has benefitted from disproportionately good resourcing it could not prevent the failure of Bristol Energy and has little appetite for long-term heat network expansion	Beyond providing value for money to their shareholders/stakeholders, Ameresco and Vattenfall dedicate most of their resources to energy infrastructure investments and service delivery	Operational and commercial risk: longer-term projects are exposed to changing investment climates and contractual inflexibility may be more susceptible to price and technological risk, as well as market, financial and political risk
Task complexity	Beyond their estate and the social housing they control, BCC has limited experience in complex energy infrastructure and service delivery projects	Ameresco and Vattenfall's have a strong track record in delivering large-scale bespoke energy service projects	Regulatory risk: regulation of heat networks might challenge connection and pricing arrangements
Market competitiveness	Through its procurement exercises, BCC has good knowledge of market competitive pricing but it will always remain a price taker	Vattenfall in particular operates at a scale that it is effectively a price maker	Monopolistic pricing risk: for privatised networks that are centrally controlled
Institutional framework	Scant government resourcing limits what local authorities can dedicate to energy infrastructure and service delivery investments	Right of first refusal'enables Ameresco and Vattenfall to scale delivery without the need for repeat procurements	Anti-trust/competition law risk: transparency needs to be upheld to ensure'right of first refusal'does not result in unreasonable restrictions on competition and price searching
Social value	BCC has an advantage in delivering social value as its creation and delivery is its core function	Ameresco and Vattenfall have experience in delivering social value as part of their contractual commitments	Performance risk: it is unclear how performance will be monitored independently and whether BCC has the contractual means to hold Ameresco and Vattenfall to the higher end of performance under the business plan

To ensure its continued success, BCC and BCL need to be transparent about how these risks are managed and if necessary mitigated. A costly outcome akin to a failed PFI which delivers infrastructure investments at much higher cost to the public sector than originally anticipated would be disastrous for the pace and scale of net zero delivery, and the overall legitimacy of such endeavours at a local level (Lewis, 2021; Sugar et al., 2022; Garvey et al., 2022, 2023). If, on the other hand, BCL delivers its KPIs, and social value in particular, to the satisfaction of independent verifiers, this ‘mode of governance’ can contribute to overcoming some of the resourcing and capability shortfalls which are preventing UK local authorities from delivering their net zero targets (Green Alliance, 2020; LGA, 2021; NAO, 2021; Sugar et al., 2022; Nolden et al., 2024). At the same time, it needs to be recognised that the BCL ‘mode of governance’ does not resolve the issue of asset ownership in the long run.

*“I think that's the difficulty with the sort of roll out of distributed networks be heat or power, it needs somebody to put in the investment in the infrastructure… Whether that ultimately should be owned by the city council, whether it should be owned by combined authorities, whether it should be owned by cooperatives… of local people, local businesses, I don’t know what the best model is for that if I’m honest. But I think that expanding that community ownership model to find ways where it can work on a bigger scale may be the way to do it.”* (#11)

*“I would argue, given the overall economic challenge of this, lots of upfront costs, relatively limited energy saving, that we don’t have room for profit extraction. So that would lend you to thinking about that [special purpose vehicle] having some sort of not-for-profit mandate. So typically, under UK company law, you’d be thinking about something like a community interest company or a company limited by guarantee so there is no equity ownership of those assets.”* (#14)

It is beyond the scope of this paper to provide an analysis of what such arrangements might look like in practice but they require further exploration to increase the social value of net zero delivery, improve legitimacy, and counter the inherent “*risk which generally public private partnerships are seen to be that if there's any money to be made it goes one way and if there's any risk it goes the other*” (#11).

## Conclusion

This research suggests that a portfolio approach to local net zero delivery, rather than a project-by-project approach, creates economies of scale which attracts investment in energy infrastructures and service delivery thanks to economies of specialisation and learning. In procuring BCL, BCC has pioneered such an approach to help achieve net zero and deliver social value alongside. Given public budget constraints, such an approach is one of the few avenues available to UK local authorities to deliver on their net zero promises. Lowering risks and transaction costs of ensuring that the delivery partner is indeed capable and willing to increase investments and deliver KPIs both at the onset and over the duration of the contract, however, requires significant resourcing. In particular, sufficient resourcing is necessary to conduct due diligence during the procurement process, to scrutinise and monitor KPI delivery independently, and to specify ‘teeth’ which apply in the case of underperformance.

BCC is in a comparatively good position to weigh-up risk and transaction costs vs investments and social value sought thanks to its extensive engagement in energy service delivery and its experience establishing and winding down an energy supply company. Yet risk remains around contract underperformance and the delivery partner cherry-picking profitable projects. Such risk is inherent in such PPPs which seek to crowd in private finance in energy infrastructure investment and service delivery. If these cannot be effectively mitigated, the legitimacy of such ‘modes of governance’ might be put into question, as they have in the past.

Overall, these findings reveal a changing risk appetite among local authorities in the delivery of their net zero ambitions. While this is welcome in principle, careful due diligence and monitoring is required to ensure that associated partnerships, agreements, and contracts secure investments, deliver KPIs, and do not fall hostage to profiteering. If these risks can be effectively mitigated, arrangements such as BCL can accelerate local net zero delivery while creating significant social value. To maximise social value delivery, however, alternative ‘modes of governance’ require exploration to avoid the inherent tension between short-term profit maximisation objectives of private investors and long-term investment requirements for a just transition to net zero.

## Data Availability

Data will be made available through the Energy Data Centre (https://ukerc.rl.ac.uk/) of the UK Energy Research Centre upon publication of the final article of the Finance and Procurement for Net Zero Research Project (https://ukerc.ac.uk/project/finance-and-procurement-for-net-zero/).

## Annex 1

KPIs (BCC, 2022a, 2022b):

- Make the Council’s own operations carbon neutral by 2025 (covering its direct energy ‘Scope 1’ and transport emissions ‘Scope 2’).
- Retrofit the Council’s social housing, which encompasses c27,500 properties and around 17% of Bristol’s housing stock, by 2030 achieving a minimum Energy Performance Certificate Band C.
- Save c. 152,000 tons of CO2.
- Deploy c. 182 MW of zero carbon energy generation.
- Deliver c. £61m of social value, which amounts to 14.4% of contract value including c. £50m of contracts to be delivered by local suppliers (BCC, 2022a, 2022b).
